# Histoplasmosis: No Longer Just a Disease of the Immunocompromised

**DOI:** 10.7759/cureus.78260

**Published:** 2025-01-30

**Authors:** Sofia M Castro, Gilberto Teixeira, Lília Andrade, João Cravo

**Affiliations:** 1 Pulmonology, Unidade Local de Saúde da Região de Aveiro, Aveiro, PRT

**Keywords:** fungal infection, hepatosplenomegaly, histoplasmosis, immunocompetent, non-endemic region

## Abstract

Histoplasmosis is a fungal infection primarily caused by the dimorphic fungus *Histoplasma capsulatum*. In endemic regions, the primary reservoirs are soil and the waste of bats and birds. The fungus spreads through the inhalation of spores in contaminated environments, often behaving as an opportunistic pathogen. Its clinical presentation is highly variable, ranging from asymptomatic cases to progressive disseminated infections, which can be fatal if left untreated. A 56-year-old man with a history of pulmonary emphysema, previous oral candidiasis, and frequent travel to Asia presented with splenomegaly, hepatosplenomegaly, leukopenia, and thrombocytopenia. An extensive diagnostic workup ruled out HIV, tropical infections, and storage diseases but revealed elevated levels of angiotensin-converting enzyme and β2 microglobulin. Imaging studies showed emphysematous changes, mediastinal adenopathy, and tree-in-bud opacities. Bronchofibroscopy identified mucosal nodular lesions, which, upon biopsy, were confirmed to be necrotizing granulomas with fungal morphology consistent with *Histoplasma*. The diagnosis of progressive disseminated histoplasmosis involving the lungs, bone marrow, and spleen was established. Treatment with intravenous amphotericin B led to clinical and radiological improvement. This case underscores the importance of considering disseminated histoplasmosis in immunocompetent patients, even in nonendemic areas. It highlights the critical role of thorough clinical history and prompt diagnosis in ensuring effective management.

## Introduction

Histoplasmosis is a fungal infection commonly caused by the dimorphic fungus *Histoplasma capsulatum* [1,2]. Endemic areas include the Ohio and Mississippi River valleys, Central and South America, and localized regions in the Eastern United States, southern Europe, Africa, and southeastern Asia [3]. The primary reservoirs of this fungus are soil and the droppings of bats and birds [3]. Infection typically occurs through the inhalation of environmental spores, and it can affect both immunocompetent and immunocompromised individuals [4], although cases of disseminated histoplasmosis are rarely reported in immunocompetent hosts [2]. Once inhaled, the spores are small enough to reach the terminal bronchioles and alveoli, where they convert to the yeast phase within macrophages [5]. Due to its mode of transmission, respiratory infection is the most common manifestation [4,5]. Histoplasmosis behaves as an opportunistic infection, with a clinical presentation that can vary widely - from asymptomatic infection to progressive disseminated disease, which can be fatal if not treated appropriately [1,6]. The mortality rate of the infection ranges from 7% to 44% [7].

This article was previously presented and discussed as a poster at the 36th Pneumology Congress and the 2nd Pneumology LUSO-PALOP Congress, held from November 12 to 14, 2020, at the EPIC SANA Algarve Congress Center in Portugal.

## Case presentation

We present the case of a 56-year-old man, fully autonomous in his daily activities, an active smoker with 40 pack-years of smoking, who reduced his consumption to just one cigarette per week. His medical history included asthma as a child, pulmonary emphysema, hepatic steatosis, dyslipidemia, elevated gamma-GT, a microadenoma of the adrenal gland, and an aortobifemoral bypass surgery in 2011 due to an extensive thrombus in the infrarenal abdominal aorta, which was associated with a small aneurysmal dilation of the same artery. He also had epididymitis in 2014 and oral candidiasis in 2016. The patient disclosed a history of heavy alcohol consumption in the past, which ceased about four years before the consultation; he now maintained sporadic alcohol intake, approximately three times a month, mostly wine and spirits. His only chronic medication was 100 mg of aspirin daily. Professionally, he was required to travel globally, mostly to Asian countries. He had no significant family history.

In 2017, he was referred to an internal medicine specialist to investigate progressive splenomegaly of unknown etiology. This had been an incidental finding in 2014 during an abdominal CT conducted as part of the workup for his dyslipidemia and elevated gamma-GT levels of 110 U/L (normal range: 9-50 U/L).

At his first consultation, his blood work revealed leukopenia (3,000 cells/µL), thrombocytopenia (40,000/µL), elevated serum angiotensin-converting enzyme (ACE) levels of 124.4 U/L (normal range: 35-90 U/L), and β2 microglobulin of 8,900 ng/mL (normal range: 800-2,400 ng/mL). His physical exam showed hepatosplenomegaly as the only significant finding. Tests for HIV, tuberculosis, Gaucher’s disease, Fabry’s disease, Niemann-Pick disease, visceral leishmaniasis, and tropical splenomegaly syndrome were all negative. A chest CT revealed emphysematous changes and multiple mediastinal adenopathy, along with additional adenopathy in the celiac, splenic, and gastric trunks. The possibility of a splenectomy was discussed but ultimately declined by the patient.

**Figure 1:**
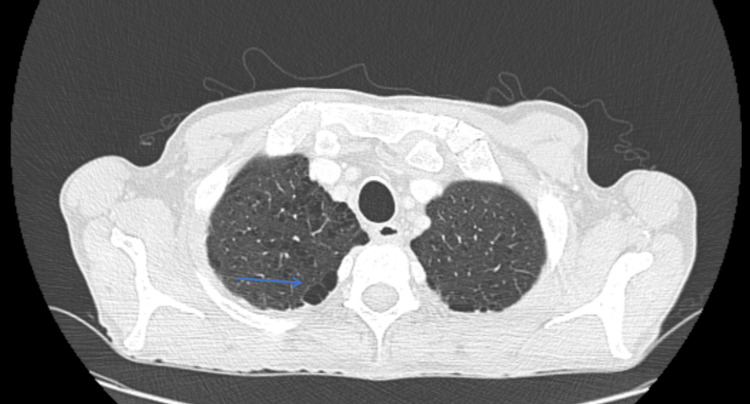
Chest CT in the axial plane showing paraseptal and centrilobular emphysema in the upper lobes, predominantly in the right lung, as indicated by the blue arrow.

**Figure 2:**
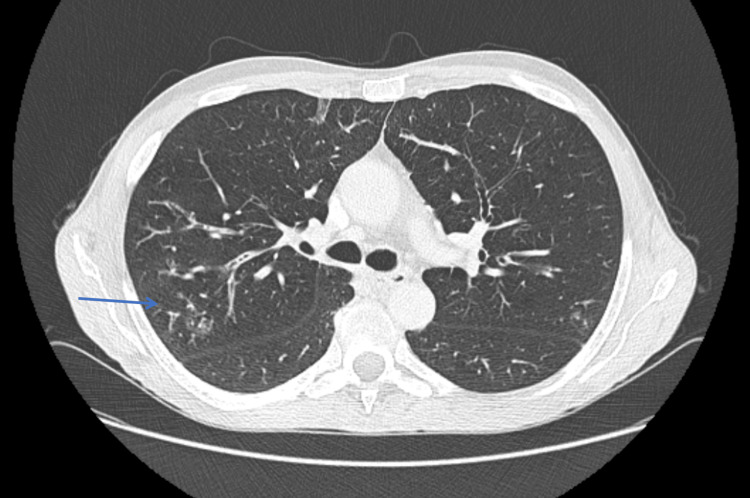
Chest CT in the axial plane showing diffuse tree-in-bud opacities, particularly in the right lung, as indicated by the blue arrow.

**Figure 3:**
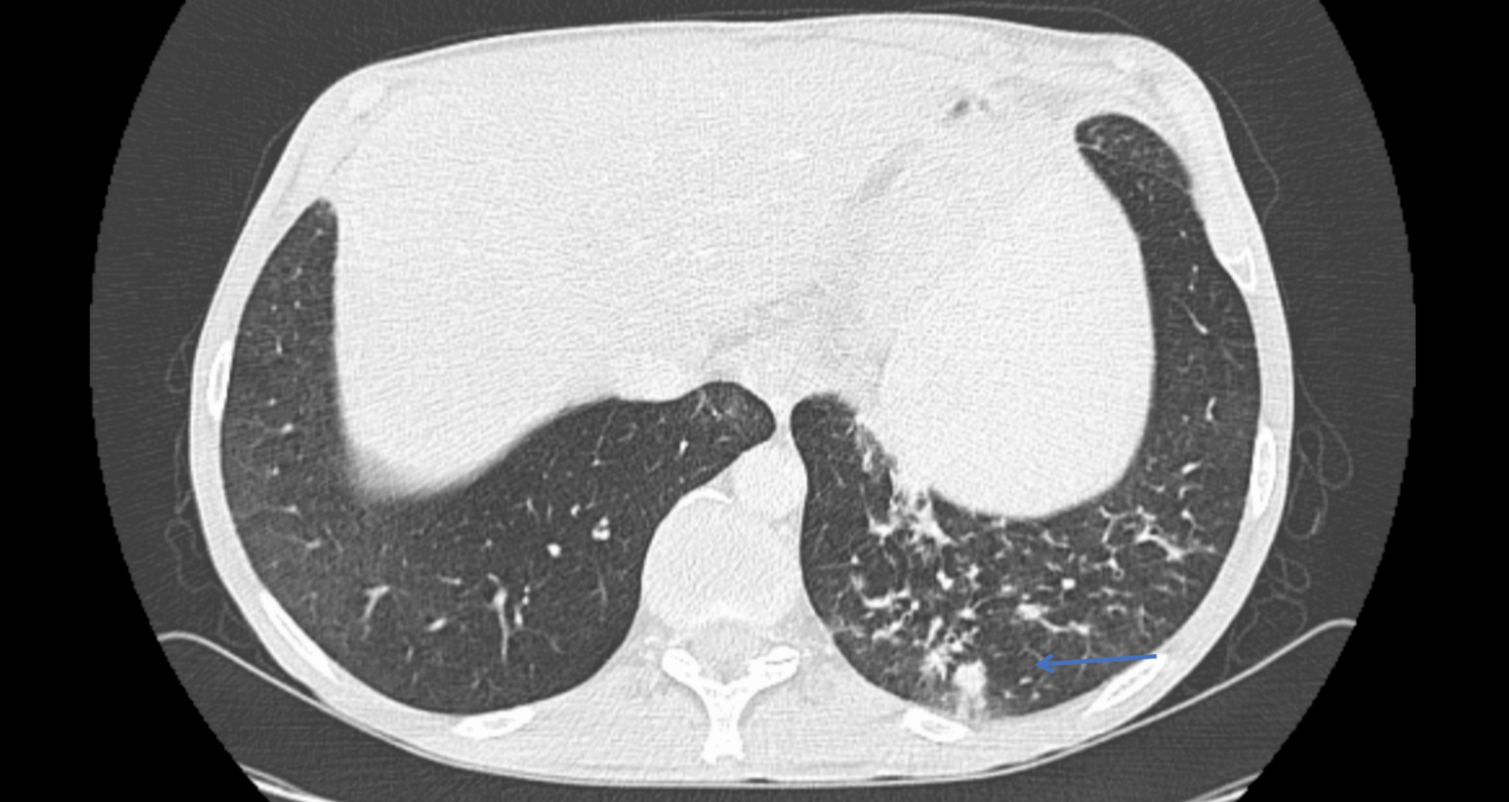
Chest CT in the axial plane showing nonspecific opacities in the left lower lobe, highlighted by the blue arrow.

In 2019, he was referred to a pulmonology outpatient clinic due to dyspnea associated with productive cough and mucopurulent sputum. Pulmonary function testing revealed obstructive respiratory disease with a positive response to bronchodilation (FEV1/FVC 53.13% predicted, FEV1 51.8% predicted, FVC 78.5% predicted). Given his persistently high ACE levels (140 U/L), a new chest CT was ordered in 2020. This CT showed diffuse tree-in-bud opacities and nonspecific opacities in the left lower lobe, along with several adenopathies in pre-vascular and pre-tracheal regions (Figure 1, Figure 2, Figure 3).

At this point, several diagnostic hypotheses were being considered, including the overlap of asthma and chronic obstructive pulmonary disease or sarcoidosis. The CT scan also revealed fibrotic streaks, which warranted close surveillance. Additional tests were performed, including the measurement of alpha-1 antitrypsin, total IgE, and allergen-specific IgE, all of which returned normal results.

**Figure 4:**
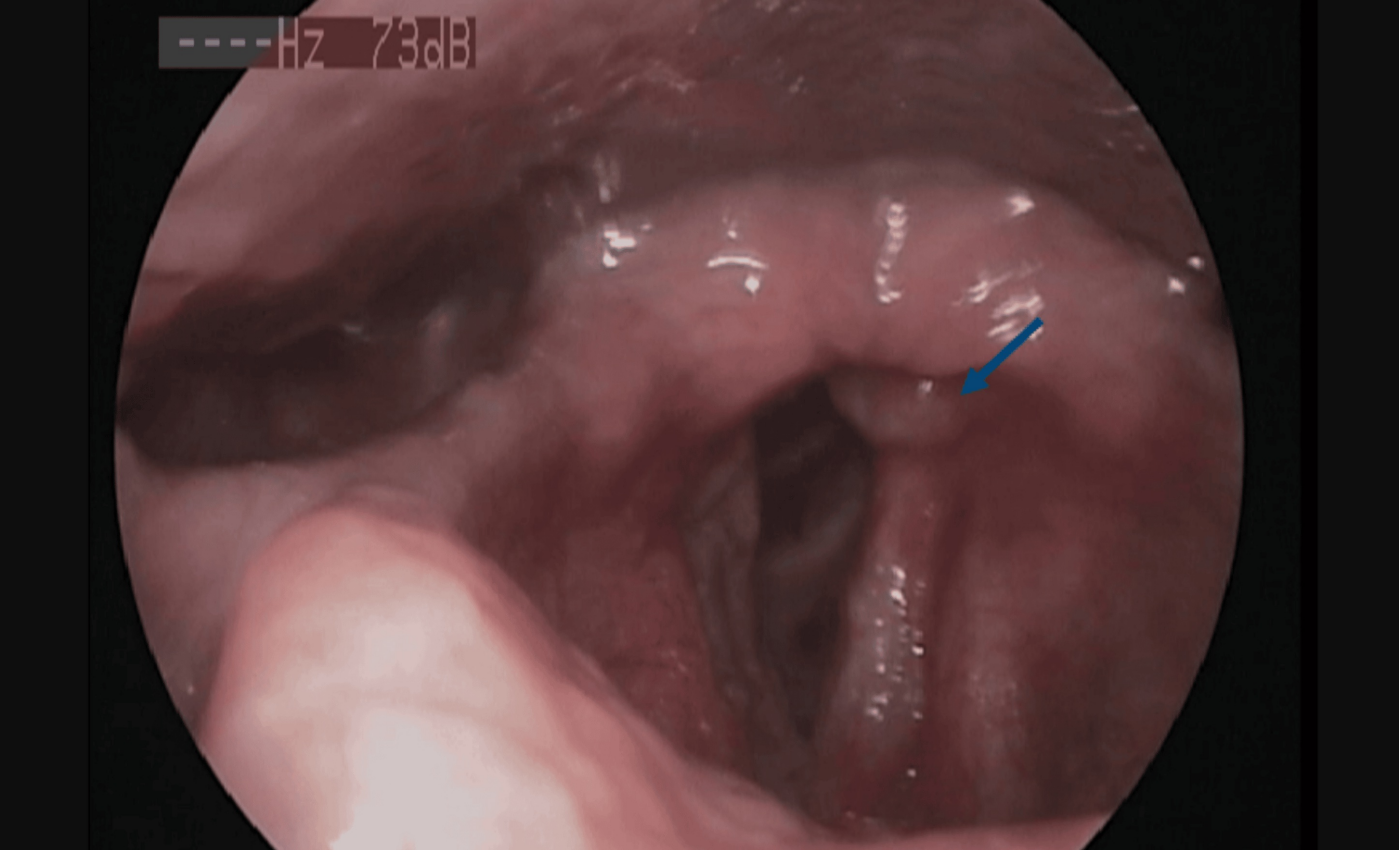
Video bronchoscopy image of the larynx and vocal cords. The blue arrow points to the nodular lesion on the right vocal cord, which was biopsied.

A flexible video bronchoscopy revealed several whitish nodular lesions in the submucosa, scattered throughout the trachea and the proximal third of the left main bronchus. Multiple biopsies were taken from the largest lesion (Figure 4). Following these findings, and as the patient developed symptoms of dysphonia and worsening dyspnea, a laryngoscopy was conducted, revealing similar nodular lesions on the right vocal cord, which were also biopsied.

Diagnosis and clinical management

**Figure 5:**
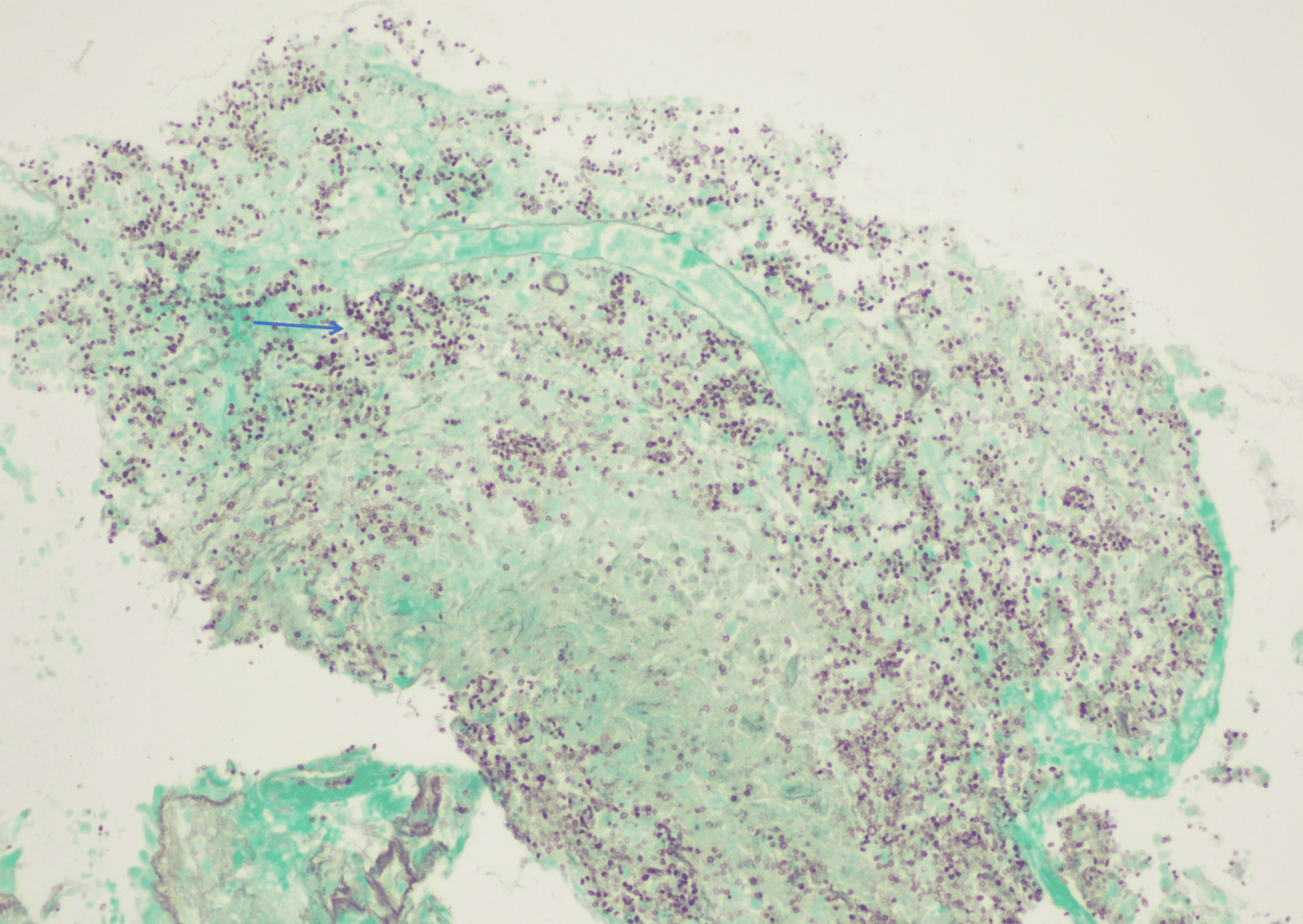
Necrotizing granuloma of the bronchial mucosa, featuring numerous fungal structures stained purple in multiple areas, with one highlighted by the blue arrow. Grocott staining, 20x magnification.

**Figure 6:**
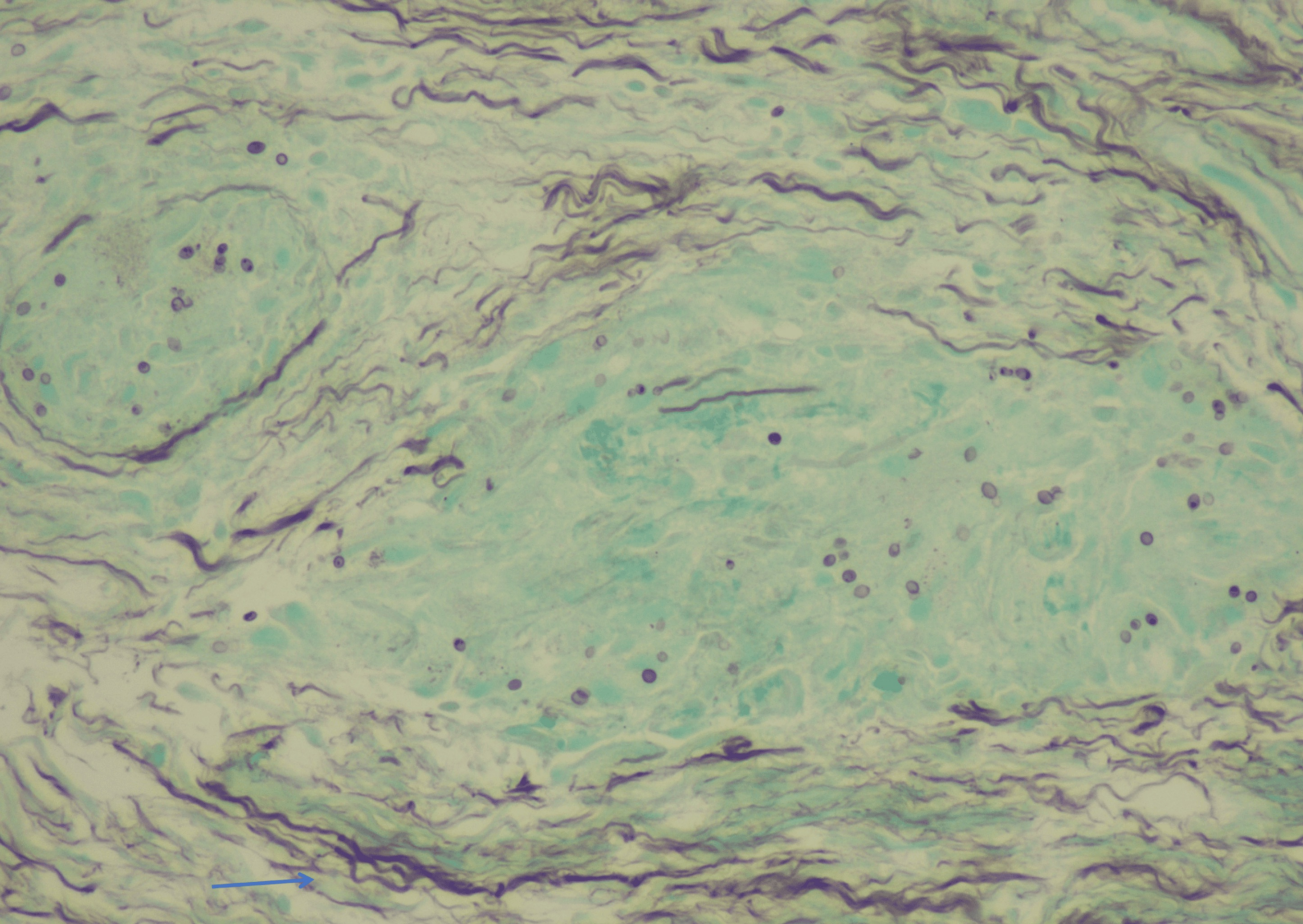
Necrotizing epithelioid granuloma of the bronchial mucosa, showing oval fungal structures with a single, narrow-based budding, stained purple. One structure is highlighted by the blue arrow. Grocott stain, 400x magnification.

Pathology results revealed that both lesions were associated with a fungal infection, with morphological and histochemical characteristics consistent with *H. capsulatum* (Figure 5, Figure 6). A review of older medical records uncovered a previous bone marrow biopsy that had revealed necrotizing granulomas. Based on these findings, a diagnosis of progressive disseminated histoplasmosis was made, considering the involvement of the lungs, pharynx, larynx, bone marrow, and associated hepatosplenomegaly.

**Figure 7:**
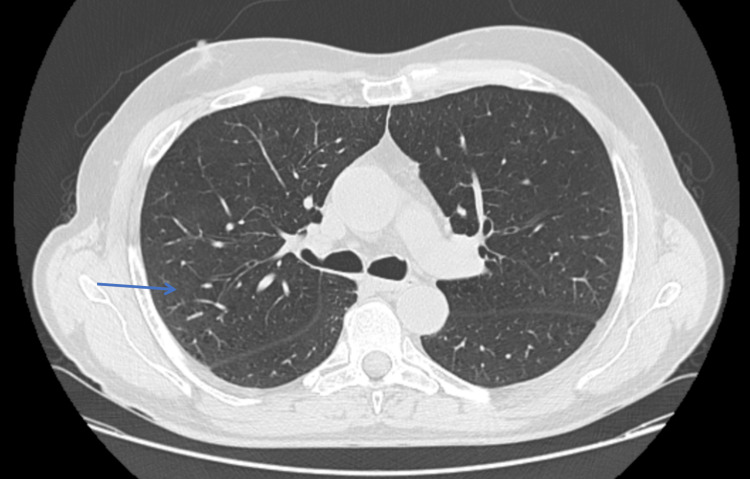
Chest CT in the axial plane showing the improvement of the diffuse tree-in-bud opacities in the area highlighted by the blue arrow.

**Figure 8:**
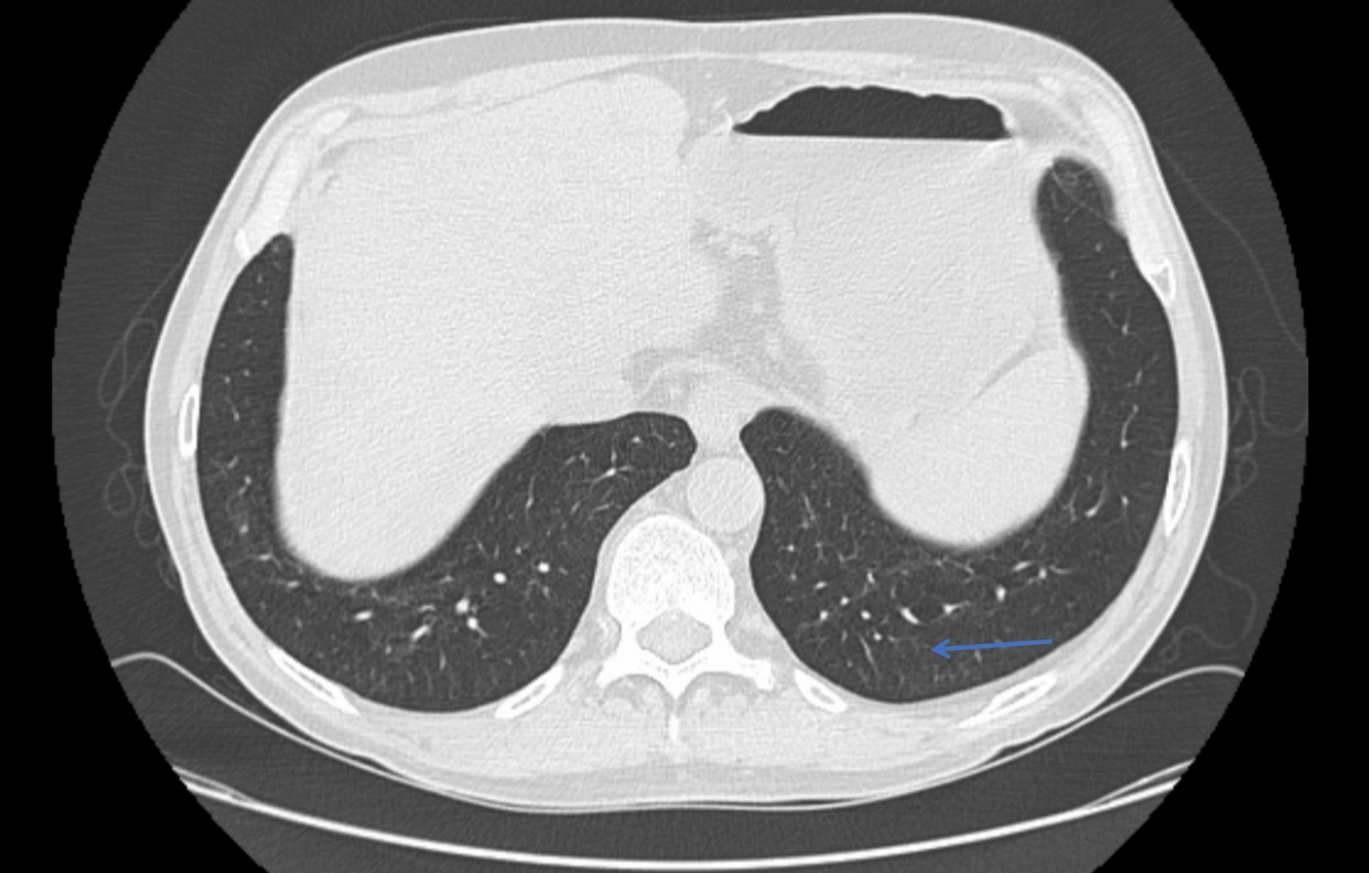
Chest CT in the axial plane showing complete resolution of the opacification in the left lower lobe. The area where it previously appeared is highlighted by the blue arrow.

He was admitted to the pulmonology department for treatment with intravenous amphotericin B, 220 mg once daily for 14 days. He responded excellently to treatment, with complete resolution of his dyspnea and cough, and was subsequently discharged. He continued treatment with oral itraconazole, starting with 200 mg three times a day for three days, followed by 200 mg twice a day for 12 months, with ongoing surveillance during pulmonology consultations. A follow-up chest CT six months into treatment showed complete resolution of pulmonary infiltrates, multiple nodular lesions, tree-in-bud markings, and adenopathies (Figure 7, Figure 8). An abdominal CT scan showed a 3 cm reduction in both liver and spleen diameters. This patient experienced significant clinical improvement and regained his voice through speech therapy.

## Discussion

This case highlights the rare occurrence of disseminated histoplasmosis in an immunocompetent individual, a phenomenon still infrequently reported in the medical literature [6]. Historically, disseminated histoplasmosis has been closely associated with immunosuppressed states, such as HIV infection or organ transplantation, and is primarily seen in endemic regions. However, emerging cases outside these populations and geographic locations challenge our traditional understanding of the disease’s epidemiology [1-3].

Cases of disseminated histoplasmosis in immunocompetent individuals have been sporadically reported in the literature, emphasizing the importance for clinicians to maintain a high index of suspicion, regardless of the patient’s immune status. For instance, Silva-Vergara et al. [1] describe a case of disseminated histoplasmosis in Brazil, highlighting the relevance of the disease in non-HIV populations within endemic areas. Similarly, a global review by the Infectious Diseases Society of America [5] identifies an increasing recognition of histoplasmosis in nontraditional patient profiles, further illustrating the expanding spectrum of the disease.

The immunological mechanisms that allow histoplasmosis to disseminate in immunocompetent patients remain under investigation, with some hypotheses suggesting subtle, undiagnosed immune deficits or a high pathogen inoculum. Kauffman [3] explored the clinical manifestations and management of histoplasmosis, emphasizing that even in immunocompetent hosts, severe presentations can occur when exposure is intense, such as through bird or bat droppings in confined spaces.

In nonendemic areas like Portugal, histoplasmosis is often not included in differential diagnoses, which can delay treatment. Emerging data, such as those reported by Singh et al. [2], emphasize the importance of considering histoplasmosis in the diagnostic workup of community-acquired pneumonia unresponsive to antibiotics, even in immunocompetent patients. Furthermore, Araj et al. [6] highlight advances in diagnostic tools, such as antigen detection, which have been crucial in facilitating early diagnosis in atypical cases.

Our case further aligns with the growing body of literature suggesting that disseminated histoplasmosis can mimic other conditions, including malignancies or other granulomatous diseases. This underscores the need for comprehensive diagnostic algorithms, as outlined by Wheat et al. [5], which incorporate imaging, histopathological examination, and molecular diagnostics to confirm the diagnosis in challenging cases.

Early diagnosis and treatment remain critical, as emphasized by Bongomin et al. [7], who show that delayed antifungal therapy in disseminated histoplasmosis is associated with worse outcomes, even in immunocompetent hosts. This finding aligns with our case, where prompt antifungal therapy played a crucial role in controlling the disease.

## Conclusions

This clinical case highlights the emerging incidence of disseminated histoplasmosis in immunocompetent patients, particularly in nonendemic areas. While disseminated histoplasmosis in otherwise healthy individuals is extremely rare, the disease is traditionally associated with immunocompromised patients, such as those with HIV, and typically presents in endemic regions. However, emerging cases worldwide indicate that histoplasmosis should now be considered in the differential diagnosis for a broader range of patients. In cases of disseminated histoplasmosis, a diagnostic algorithm may be employed. When faced with isolated community-acquired pneumonia of unknown etiology that is unresponsive to antibiotics or accompanied by hepatosplenomegaly, lymphadenopathy, and pancreatic involvement; significant exposure to bird or bat droppings; imaging studies revealing pulmonary nodules or lymphadenopathy; or a known connection to a histoplasmosis outbreak, histoplasmosis should be strongly considered as a potential diagnosis.

Early recognition and prompt initiation of appropriate antifungal therapy are essential for improving outcomes. Clinicians should maintain a high index of suspicion for histoplasmosis, regardless of a patient’s immune status or geographic location, to ensure timely diagnosis and treatment, which can effectively control the disease in most cases.
